# Plant Invasion-Induced Habitat Changes Impact a Bird Community through the Taxonomic Filtering of Habitat Assemblages

**DOI:** 10.3390/ani14111574

**Published:** 2024-05-25

**Authors:** Emilia Grzędzicka

**Affiliations:** Institute of Systematics and Evolution of Animals of the Polish Academy of Sciences, Sławkowska Street 17, 31-016 Kraków, Poland; grzedzicka@isez.pan.krakow.pl

**Keywords:** bird community, beta diversity, species richness, plant invasion, agricultural landscape

## Abstract

**Simple Summary:**

Simple Summary: This research focused on the taxonomic filtering of terrestrial birds in plant invasion-altered landscapes. There were assessed changes in bird habitat assemblages (ground/herb dwellers, bush foragers, ecotone birds, and tree foragers) associated with a gradient of invasion on sites (far from the invasion, uninvaded although susceptible to invaders, and invaded) set in European agricultural lands. The results indicated the impact of invasion on two habitat assemblages of birds (i.e., ground/herb species loss and creating the distinctive bush species composition) and a decrease in the abundance and species diversity of birds from the whole community in the invaded areas.

**Abstract:**

Describing the spatial distribution of communities is crucial to understanding how environmental disturbance can affect biodiversity. Agricultural lands are susceptible to disturbances of anthropogenic origin and have been identified as ecosystems of conservation concern. Such lands are vulnerable to invasions by anthropogenically introduced non-native plants disturbing habitats. This research focused on the invasion-induced taxonomic filtering of birds with shared habitat requirements. The birds were surveyed along a gradient of invasion-altered areas (far from the invasion, uninvaded although susceptible to invaders, and invaded) to identify changes in bird assemblages (ground/herb dwellers, bush foragers, ecotone birds, and tree foragers) caused by this disturbance. Data were collected from 112 sites sampled in southeastern Poland. There were significantly fewer bird species from each assemblage on invaded sites than on uninvaded sites, although exposed to invasion, despite the decrease in the abundance of only ground/herb dwellers. Beta diversity analysis showed that sites with invasion contained bird communities significantly different from those at other sites. Invasion-induced changes resulted in a significant reduction in the diversity of ground/herb dwellers in comparison with uninvaded sites and created a distinctive bush bird assemblage. This was most likely due to the transformation of the grassland layer and the thickening of the shrub layer by plant invaders, which resemble shrubs in morphology. The results indicated the filtering effect of invasion on bird species composition at the level of two habitat assemblages despite the decreases in bird abundance and species diversity of the whole community.

## 1. Introduction

Landscape changes caused by human activities are the main cause of the current biodiversity crisis [1,2,3,4]. The scale and extent of these activities create specific anthropogenic ecosystems [5]. On the other hand, land-use diversity is an important environmental factor that promotes species richness treated as a measure of local alpha diversity. However, understanding processes that shape the wider measure of species composition among different sites (beta diversity) remains a fundamental challenge for ecologists [6]. Assessing the relationship between land use and both measures (alpha, beta) of regional diversity can affect our understanding of global biodiversity patterns [7].

Agricultural landscapes contain areas of various land use and habitats. There is a lack of research on the responses of animals to habitat changes in such landscapes and whether their habitat requirements impact those responses filtering the community. The concept of environmental filtering assumes that the occurrence, function, and survival of a species at a particular site is driven by its tolerance or adaptation to environmental conditions [8,9], and various species differ in this tolerance. The measured variation in species composition between habitats (or sites) is the definition of beta diversity [10]. Assessing beta diversity can be understood as studying variation in species among sites linking biodiversity at local scales (alpha diversity) and the broader regional species pool (gamma diversity) [11,12]. Alpha measures how diversified the species are within a site, whereas beta measures how diversified the sites are in species composition within a region [11,13,14]. Beta diversity has become a sophisticated concept [14,15,16].

Beta diversity can be assessed through the extent of species compositional differences between sites to detect the assembly mechanisms [10]. The division of beta diversity into two components, turnover and nestedness, is recommended. Turnover assumes that species are replaced by different ones comparing one site to the next (i.e., species replacement), whereas nestedness patterns appear when species loss or gain causes species-poor sites to resemble a strict subset of species-rich sites [10,17,18]. It is worth emphasizing that replacement and richness difference or nestedness indices should be enriched with a careful ecological interpretation [18]. Turnover may result from species endemism at various spatial scales and does not take into account that similar species may be spatially exclusive due to their properties or have specific range boundaries [19]. Nestedness may result from local extinctions or colonization [20]. Therefore, spatial variation in the composition of species must take into account the temporal aspect, because the current community is only its temporary version and may be the result of deterministic and/or stochastic processes [21]. Deterministic processes result from habitat properties (e.g., habitat filtering or competitive interactions for resources) in determining the composition of local communities [10]. Stochastic processes are local colonization or extinction that are the responses of species to processes outside the studied environment [21].

In the case of mobile animals such as birds, which respond rapidly to changes in habitats, examining nestedness and turnover separately may be misleading. For example, species nestedness accounted for a small proportion of bird beta diversity in Sri Lanka’s biodiversity hotspot. Bird community assembly was, on the other hand, strongly influenced by climate at a surprisingly small scale [22]. The filtering effect of vegetation is sometimes independent of species losses and replacements, although often remains a key predictor of their beta diversity [9]. In line with this phenomenon, vertically developed vegetation affected bird assemblages through niche partitioning from ground to tree canopies in forests, and the largest independent effect concerned canopy height [23,24]. Although individual layers of the forest contain different bird assemblages, it is difficult to expect that this type of variability of species in space reflects beta diversity, because it is located in one type of habitat. In contrast, the positive relationship between habitat heterogeneity [9] and beta diversity is highly detectable in studies investigating the environmental/habitat heterogeneity hypothesis. Since decisions and the range of occurrence of such small taxonomic groups as species may be the result of habitat filtering rather than relationships between species at different sites following the idea of beta diversity, it seems reasonable to use the concept of beta diversity when comparing broader and ecologically related groups, e.g., several species choosing a similar habitat.

Investigating determinants of the diversity of groups is important, as in a biodiversity crisis, not only are specific species extinct, but entire communities associated with specific environments are at risk. A large number of studies explored bird diversity in urban areas [25], agricultural lands [26,27], and forests [28]. Biodiversity loss has been a disproportionally ongoing problem in open landscapes such as grasslands, agricultural lands, and steppes in Europe in the last few decades [29]. Such declines are believed to be driven by agricultural intensification and subsequent habitat disturbances [30] as well as by the abandonment of agricultural land [31]. The relationships between habitats and community diversity in agricultural landscapes may be critical to the survival of species and biodiversity across a wide range. However, the patterns of population changes were hardly detectable when based on species using agricultural lands without more precise habitat discriminations [32]. This is the reason why the present article examines the diversity of birds sorted into assemblages connected with the vegetation layers from the ground to the tree canopy [23,24]. This research investigated changes in the following bird assemblages: ground/herb dwellers, bush foragers, ecotone birds, and tree foragers. This was in line with the fact that the vertical composition of vegetation located in a mosaic of habitats can be a driver of the birds’ beta diversity patterns [23,24]. 

However, bird surveys comparing paired sites, for example, controlled vs. disturbed, in the same habitats and landscape heterogeneity are not sufficient to understand the patterns of bird diversity change driven by this disturbance because these paired sites are in some way selected. It is impossible to determine which bird species do not reach the site because they do not appear there due to the location or heterogeneity. Therefore, birds at reference sites (easily available for the studied bird assemblages, although not exposed to disturbance, i.e., far from the landscape heterogeneity of the paired sites) were analyzed with the expectation that they constituted a wider bird community than the paired sites.

As mentioned, agricultural lands are exposed to various anthropogenic disturbances, such as plant invasions. This research evaluates the taxonomic filtering of bird assemblages along a gradient of plant invasion-altered agricultural lands. The focal invaders were Caucasian hogweeds or *Heracleum* sp., which were once introduced to be used as crops. These invaders, spreading unpredictably and reaching four meters in height, were assumed to be independent from the turnover and nestedness patterns in line with the fact that in other research, the largest independent effect concerned canopy height [23,24]. It has not been studied whether and how invasive plants affect a bird community in the context of the beta diversity concept. The aim of this article is to fill this knowledge gap.

The selected invaders resemble bushes in physiognomy and reach large sizes, so it was predicted that by degrading the habitat, they may have had a significant negative impact on the birds from all assemblages, but may have created new space for bush foragers or ecotone species using bushes. In line with the idea of beta diversity, the composition of these assemblages can differ on invaded sites when compared to uninvaded ones. Assuming that the more individuals there are in an assemblage, the more species there are in its composition, variability was also assessed in the bird abundances of individual assemblages between the sites. This was intended to investigate the possible problem of smaller differences between the sites in alpha and beta diversity in the more widely represented assemblages.

## 2. Materials and Methods

### 2.1. Invading Plant Species

The invaders were species of hogweeds, giant hogweed *Heracleum mantegazzianum* and Sosnowsky’s hogweed *H. sosnowskyi*, both originating from the Caucasus region. They were introduced to several central and eastern European countries to be used as crops and melliferous plants. Starting from the late 1980s and early 1990s, many Caucasian hogweeds’ plantations were abandoned. Nowadays, they are widely spread along river valleys, roads, and railway lines mainly in the European continent and North America [33].

### 2.2. Study Sites

This research was conducted in southeastern Poland on *n* = 112 sites set as 28 pairs and the accompanying references for each site. Within each pair, one site contained Caucasian hogweeds (the site hereafter called “*Heracleum*”), while the invaders were absent at the second site (“control”). Each study site was a circle with a radius of 100 m (i.e., covering 3.14 ha). Study sites with hogweeds were set in localities, where it was possible to determine the radius of 100 m from the planned bird surveying point without buildings and busy roads. Control sites were randomly selected by choosing the area with a similar habitat mosaic as those with invasion, but not closer than 500 m from the *Heracleum* site centers to avoid double surveys of the same birds at different points. Both points from each pair were set in the same region, with a similar habitat and landscape heterogeneity. 

The assumption of bird surveys on reference sites was to complement the knowledge about local bird fauna in a given region, because some species may have been omitted during surveys on pairs of sites as not using a specific heterogeneity or habitats present on them. The reference sites contained habitats that could potentially be selected by the birds present on the accompanying paired sites. Reference sites were distant from the linear and anthropogenic elements near the paired sites, which could facilitate invasion but also increase habitat heterogeneity. For example, in river valleys, reference sites contained similar habitats (i.e., open or overgrown, respectively) to those along the river, although farther from the river and countryside. The site marked as a reference for the invaded site was easily available for birds from a given invaded site, while the reference for the control site was easily available for birds living on the control site. It was assumed that bird fauna from both reference sites provided a complete view of the bird community in habitats that could have occurred on paired invaded or uninvaded sites without linear and anthropogenic elements affecting both the invasion and birds.

The distance between site centers within a given pair (*Heracleum*, control) ranged from 540 m to 6 km and the distances of adjacent pairs were from 550 m to 70 km. The distance between invaded or control sites and the respective references ranged from 597 m to 3.4 km.

### 2.3. Bird Surveys

Birds were surveyed at all study sites (controls, *Heracleum*, and references). On each site, the birds were observed on three dates—first, when Caucasian hogweeds were just visible early in the season, while the second and third dates were chosen when Caucasian hogweeds were developed and could affect birds (1st survey: 19–30 April 2021; 2nd survey: 6–21 May 2021 before hogweeds’ flowering; 3rd survey: 1–24 June 2021 during the flowering period), with a minimum interval of 14 days between surveys on a given site. All sites were initially set the year before (2020) to have a view of the local topography and distribution of invaders in a given region, which gave the possibility of surveying birds quickly and correctly starting from the earliest dates in 2021. On the earliest surveys, some migrating birds may not have been observed yet, but there was a peak in activity of several resident species, which was the reason for the timing of the surveys. This assumption was crucial for the maximum species and abundance detectability. At invaded sites, birds used both native vegetation and invaders’ patches (personal observations from 2019 to 2024), so the differences between invaded and other sites were not due to bypassing invaders by birds nor reducing the available space, but resulted from their various responses. 

During the surveys, the observer was standing in the center of the study site and recorded all birds seen and heard within a radius of 100 m for 10 min. After the 10 min. survey, the observer approached the edge of the 100 m circle and performed supplementary counting for an additional 10 min when moving from edge to edge through the center of the circle. It was noted that the moving observer aroused the birds’ reaction, which contributed to their detection. The surveys were performed from sunrise to 11 a.m.

The maximum count for each species was considered as its abundance at a study site Birds that were not considered included aerial feeders, birds associated with water, urban breeders, raptors, and birds flying over the sites, as these species could blur the intention to detect which birds appeared on reference sites and not on more anthropogenic paired sites (i.e., *Heracleum* vs. control). In total, 80 species were considered for further analyses.

To perform the analyses, all bird species were sorted into four assemblages based on their nesting and foraging niches in the breeding and early post-breeding season: (1) ground/herb dwellers; (2) bush foragers; (3) ecotone birds; and (4) tree foragers. For this classification, the author used the literature [34,35] and personal experience based on observations during fieldwork.

### 2.4. Statistical Analysis

All statistical analyses were prepared in R 4.0.4 [36]. Before starting the analyses, the data were tested for a lack of outliers by preparing scatter plots between habitat variables (meadows, forested areas, etc.) and abundances of birds from particular assemblages. Using the Shapiro–Wilk test, it was shown that all habitat variables were not normally distributed. To assess whether the predicted impacts of habitats on bird assemblages between invaded, control, and reference sites did not result from the differences between areas of particular habitats, habitat variables and a number of patches reflecting habitat heterogeneity on sites were compared using the non-parametric Kruskal–Wallis test. The post hoc Dunn test was used to investigate differences in habitat areas and the number of patches by comparing sites from the three groups (control vs. invaded vs. references—Appendix A). In this test, *p*-values were adjusted using the Benjamini–Hochberg method.

To test the prediction of comparable lists of bird species detectable on control and invaded sites in contrast with a potentially wider community on the reference sites and, therefore, to investigate differences between bird communities at the control, invaded, and reference sites, the “vegan” package [37] was chosen to compare groups via a Detrended Correspondence Analysis (DCA). The natural logarithm of 1 + arg was computed based on the species matrix using the decorana function. Species were grouped using the nominal variable—“group ID” (control, invaded, references)—and used to prepare a graph with the ordihull command. The positions of species along DCA1 and DCA2 expressed with name abbreviations were based on the species scores. The extent of communities from each group of sites based on the distance across sites was illustrated in the accompanying DCA plot and expressed by the polygon option.

The Shapiro–Wilk test was used to investigate that the total abundance and species diversity, as well as the abundance and species richness of ground/herb dwellers, ecotone birds, bush foragers, and tree birds, which were in most cases not normally distributed (*p* < 0.001). Such data were normalized in the “bestNormalize” package [38,39], according to the transformation method chosen by this package. Then, the data were tested with Bartlett’s test for homogeneity of variances in the “car” package [40] and the data means were compared between the types of sites (invaded, control, references) using the One-way ANOVA test (when the test result was insignificant) or Welch ANOVA (if the test gave a significant result) and post hoc Tukey test using the “dplyr” [41] and “PMCMRplus” [42] packages.

The overall beta diversity of each bird assemblage was calculated to evaluate the relative importance of the invasion-altered site gradient in creating dissimilarity of given assemblages among sites. For this analysis, the author used the pairwise Jaccard dissimilarity index with the function beta.pair (R script in Appendix B) within the “betapart” package [43]. Differences between sites in distances from the centroid were compared with One-way ANOVA and Tukey’s post hoc test.

## 3. Results

During this research, the author noted 80 bird species actively using habitats on study sites; 72 species (1698 bird individuals) were recorded on *n* = 28 control sites, 65 species (1272 bird individuals) on *Heracleum* sites (*n* = 28), as well as 70 species (2928 bird individuals) on *n* = 56 reference sites. Taking into account the classification of birds into particular assemblages, there were 427 observed ground/herb dwellers, 365 bush foragers, 362 ecotone birds, and 521 tree foragers on control sites; on *Heracleum* sites, 232 ground/herb dwellers, 362 bush foragers, 252 ecotone birds, and 397 tree foragers; while on the reference sites, 843 ground/herb dwellers, 598 bush foragers, 546 ecotone birds, and 906 tree foragers. The control sites were dominated by the common starling *Sturnus vulgaris*—ecotone bird, skylark *Alauda arvensis*—ground/herb dwelling species, and fieldfare *Turdus pilaris*—ecotone bird, while the invaded sites hosted a large abundance of common whitethroat *Sylvia communis*—bush forager, common starling—ecotone bird, and great tit *Parus major*—tree forager. On the reference sites, the three dominating bird species were the following: skylark—ground/herb dweller, common starling—ecotone species, and great tit—tree forager.

**Figure 1:**
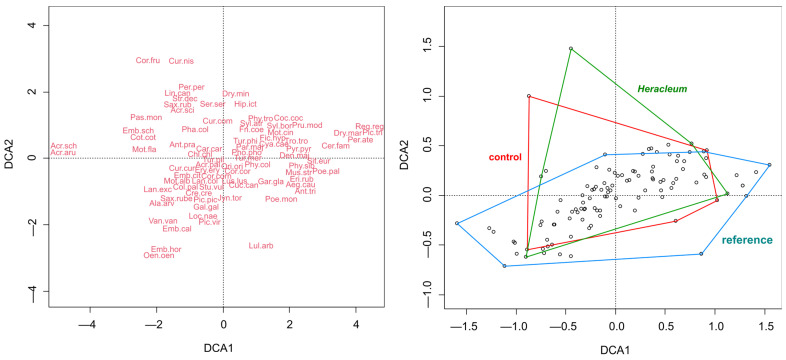
Results of the Detrended Correspondence Analysis (DCA) based on the bird community detected on *n* = 112 study sites grouped as control, invaded (i.e., *Heracleum*), and reference sites. The full Latin names of species consist of the following: Cot.cot—*Coturnix coturnix*, Pha.col—*Phasianus colchicus*, Per.per—*Perdix perdix*, Col.pal—*Columba palumbus*, Str.dec—*Streptopelia decaocto*, Cuc.can—*Cuculus canorus*, Cre.cre—*Crex crex*, Gal.gal—*Gallinago gallinago*, Van.van—*Vanellus vanellus*, Jyn.tor—*Jynx torquilla*, Pic.vir—*Picus viridis*, Dry.mar—*Dryocopus martius*, Den.maj—*Dendrocopos major*, Dry.min—*Dryobates minor*, Pic.tri—*Picoides tridactylus*, Ori.ori—*Oriolus oriolus*, Lan.col—*Lanius collurio*, Lan.exc—*Lanius excubitor*, Gar.gla—*Garrulus glandarius*, Pic.pic—*Pica pica*, Cor.fru—*Corvus frugilegus*, Cor.cor—*Corvus corax*, Cor.corn—*Corvus cornix*, Per.ate—*Periparus ater*, Poe.pal—*Poecile palustris*, Poe.mon—*Poecile montanus*, Cya.cae—*Cyanistes caeruleus*, Par.maj—*Parus major*, Lul.arb—*Lullula arborea*, Ala.arv—*Alauda arvensis*, Loc.nae—*Locustella naevia*, Hip.ict—*Hippolais icterina*, Acr.pal—*Acrocephalus palustris*, Acr.sci—*Acrocephalus scirpaceus*, Acr.aru—*Acrocephalus arundinaceus*, Acr.sch—*Acrocephalus schoenobaenus*, Phy.sib—*Phylloscopus sibilatrix*, Phy.tro—*Phylloscopus trochilus*, Phy.col—*Phylloscopus collybita*, Aeg.cau—*Aegithalos caudatus*, Syl.atr—*Sylvia atricapilla*, Syl.bor—*Sylvia borin*, Syl.nis—*Sylvia nisoria*, Syl.cur—*Sylvia curruca*, Syl.com—*Sylvia communis*, Reg.reg—*Regulus regulus*, Sit.eur—*Sitta europaea*, Cer.fam—*Certhia familiaris*, Tro.tro—*Troglodytes troglodytes*, Stu.vul—*Sturnus vulgaris*, Eri.rub—*Erithacus rubecula*, Lus.lus—*Luscinia luscinia*, Fic.hyp—*Ficedula hypoleuca*, Mus.str—*Muscicapa striata*, Pho.pho—*Phoenicurus phoenicurus*, Sax.rube—*Saxicola rubetra*, Sax.rub—*Saxicola rubicola*, Oen.oen—*Oenanthe oenanthe*, Tur.phi—*Turdus philomelos*, Tur.mer—*Turdus merula*, Tur.pil—*Turdus pilaris*, Pru.mod—*Prunella modularis*, Pas.mon—*Passer montanus*, Ant.tri—*Anthus trivialis*, Ant.pra—*Anthus pratensis*, Mot.fla—*Motacilla flava*, Mot.cin—*Motacilla cinerea*, Mot.alb—*Motacilla alba*, Fri.coe—*Fringilla coelebs*, Coc.coc—*Coccothraustes coccothraustes*, Ery.ery—*Erythrina erythrina*, Pyr.pyr—*Pyrrhula pyrrhula*, Chl.chl—*Chloris chloris*, Lin.can—*Linaria cannabina*, Car.car—*Carduelis carduelis*, Ser.ser—*Serinus serinus*, Emb.cal—*Emberiza calandra*, Emb.hor—*Emberiza hortulana*, Emb.cit—*Emberiza citrinella*, Emb.sch—*Emberiza schoeniclus*.

Birds appearing on control or invaded sites were part of a set of particular species from the wider community present on the reference sites (Figure 1). These reference sites contained habitat specialists, like the ortolan bunting *Emberiza hortulana* living in agricultural lands near young forests, northern wheatear *Oenanthe oenanthe* requiring groups of rocks in agricultural mosaics, as well as wetland birds, like the sedge warbler *Acrocephalus schoenobaenus* and great reed warbler *Acrocephalus arundinaceus*. On the reference sites, large woodpeckers typical of mature forests were also present, for example, the Eurasian three-toed woodpecker *Picoides tridactylus* and black woodpecker *Dryocopus martius*. Although on control and invaded sites many low-demanding species appeared and they were shared with the reference sites, bird species like the rook *Corvus frugilegus* or barred warbler *Sylvia nisoria* found more appropriate habitats on those paired sites in comparison with the reference ones.

**Figure 2:**
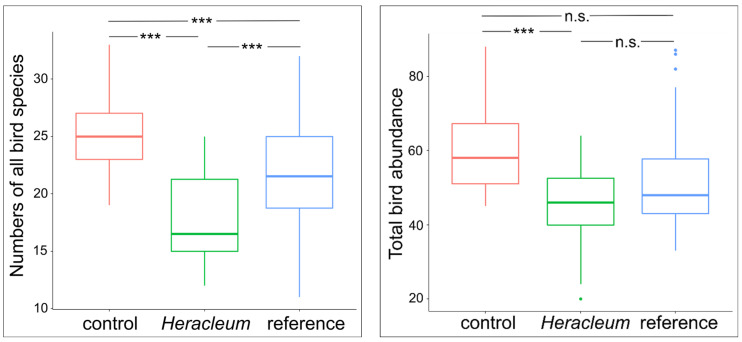
Results of post hoc Tukey test showing differences in total species richness and abundance of birds between sites (*p*-values: *** < 0.001, n.s.—non-significant).

The variance in the number of species of all birds significantly differed between the sites (Bartlett’s test = 6.876; *p* = 0.032), and there were also differences between the means (Welch ANOVA: F = 27.761; *p* < 0.001). There were significant differences found in the species richness of all birds between the *Heracleum* and control sites (Tukey’s HSD, *p* < 0.001), control vs. reference sites (*p* = 0.004), and *Heracleum* vs. reference sites (*p* < 0.001)—Figure 2. The variance in the abundance of all birds did not differ between sites (Bartlett’s test = 3.031; *p* = 0.219), although the means were different (One-way ANOVA: F = 5.638; *p* = 0.005). This resulted from the differences between the *Heracleum* and control sites (Tukey’s HSD, *p* = 0.003), as controls did not differ from references (*p* = 0.133) and there were no differences between the *Heracleum* sites and references (*p* = 0.133)—Figure 2.

**Figure 3:**
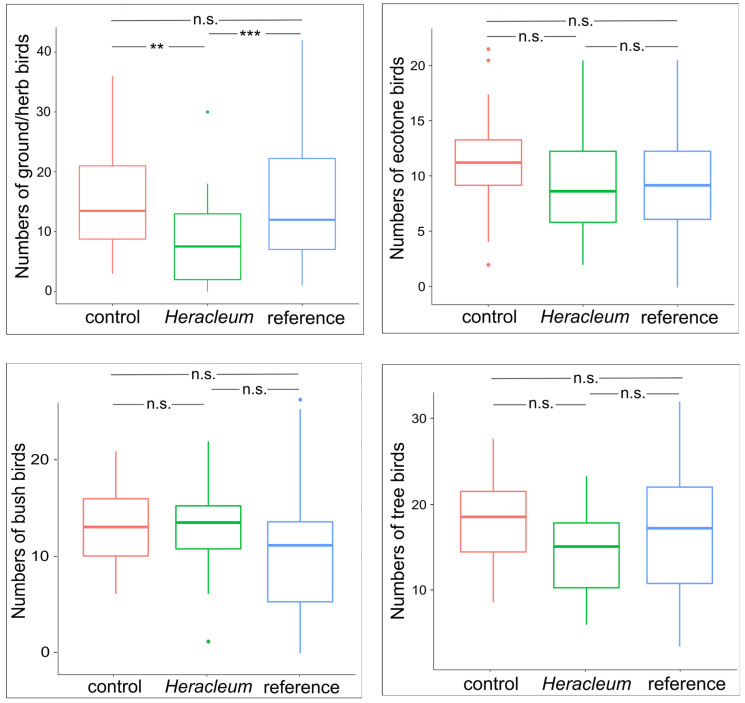
Results of post hoc Tukey test showing differences in bird abundance from each assemblage between sites (*p*-values: ** < 0.01, *** < 0.001, n.s.—non-significant).

In the case of the abundance of ground/herb birds, there were no differences in the variances between the sites (Bartlett’s test = 4.487, *p* = 0.106), but differences in the means between them were found (One-way ANOVA: F = 5.952, *p* = 0.003), resulting from differences between the *Heracleum* and control sites (Tukey’s HSD, *p* = 0.009) and reference and *Heracleum* sites (Tukey’s HSD, *p* = 0.005). There were no differences between the controls and references (Tukey’s HSD, *p* = 0.995)—Figure 3. No differences in variance were found in the number of ecotone birds between sites (Bartlett’s test = 0.277, *p* = 0.871), similarly as in their means (One-way ANOVA: F = 1.529, *p* = 0.222). In this assemblage, there were no differences between the control and *Heracleum* sites (*p* = 0.239), and no differences were found between the remaining sites (controls vs. references: *p* = 0.298; *Heracleum* vs. references: *p* = 0.926). In the case of a number of bush birds, differences in variance between sites were significant (Bartlett’s test = 11.309, *p* = 0.003), although no differences were found between the means (Welch ANOVA: F = 2.479, *p* = 0.091), which resulted from the lack of differences between the control and *Heracleum* sites (*p* = 0.989) and no differences between the remaining sites (controls vs. references: *p* = 0.161; *Heracleum* vs. references: *p* = 0.180). There were significant differences in the variances of the numbers of tree birds between sites (Bartlett’s test = 8.767, *p* = 0.012) and between the means (Welch ANOVA: F = 4.304, *p* = 0.017). There were no differences between the mean abundances of tree birds at the control and *Heracleum* sites (*p* = 0.057) and no differences between the remaining sites (controls vs. references: *p* = 0.308; *Heracleum* vs. references: *p* = 0.449)—Figure 3.

**Figure 4:**
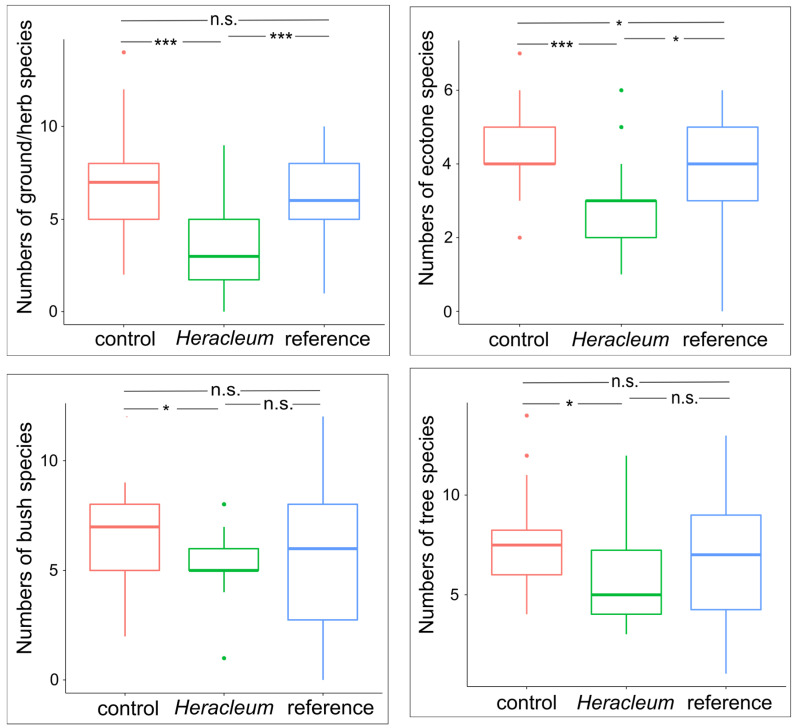
Results of post hoc Tukey test showing differences in species richness of birds from each assemblage between sites (*p*-values: * < 0.05, *** < 0.001, n.s.—non-significant).

In the case of the number of ground/herb species, there were no differences in the variances between the sites (Bartlett’s test = 1.165, *p* = 0.558), but differences in the means between them were found (One-way ANOVA: F = 10.56, *p* < 0.001), resulting from differences between the *Heracleum* and control sites (Tukey’s HSD, *p* < 0.001) and references and *Heracleum* (Tukey’s HSD, *p* < 0.001) but no differences between controls and references (Tukey’s HSD, *p* = 0.455)—Figure 4. No differences in variances were found in the number of ecotone species between sites (Bartlett’s test = 3.627, *p* = 0.163), but the differences in the means were significant (One-way ANOVA: F = 6.876, *p* < 0.001) and resulted from differences between the control and *Heracleum* sites (*p* < 0.001), although no differences were found between the remaining sites (controls vs. references: *p* = 0.058; *Heracleum* vs. references: *p* = 0.126). In the case of a number of bush species, differences in variance between sites were significant (Bartlett’s test = 18.982, *p* < 0.001), as were differences between the means (Welch ANOVA: F = 3.8, *p* = 0.027), which resulted from differences between the controls and *Heracleum* sites (*p* = 0.045) and no differences between the remaining sites (controls vs. references: *p* = 0.086; *Heracleum* vs. references: *p* = 0.991). There were significant differences in the variances of the numbers of tree bird species between sites (Bartlett’s test = 7.371, *p* = 0.025) and between the means (Welch ANOVA: F = 3.609, *p* = 0.032). There were differences between the mean numbers of tree species between the controls and *Heracleum* sites (*p* < 0.038), although there were no differences between the remaining sites (controls vs. references: *p* = 0.552; *Heracleum* vs. references: *p* = 0.325)—Figure 4.

**Figure 5:**
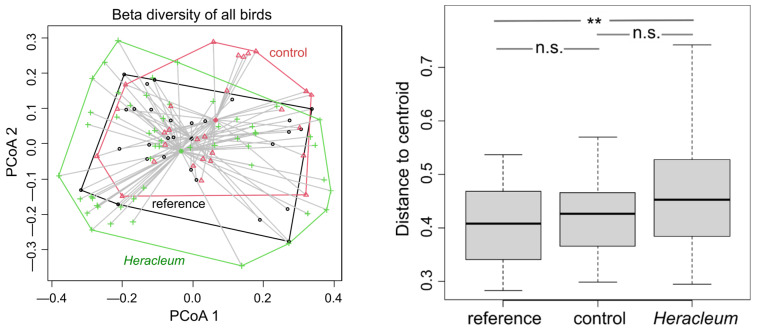
Scatter plot depicting the first two axes of the ordinations of all bird species compositions and boxplots showing the distances of each site to the centroid, reflecting differences between paired and reference sites in species compositions (*p*-values: ** < 0.01, n.s.—non-significant).

**Figure 6:**
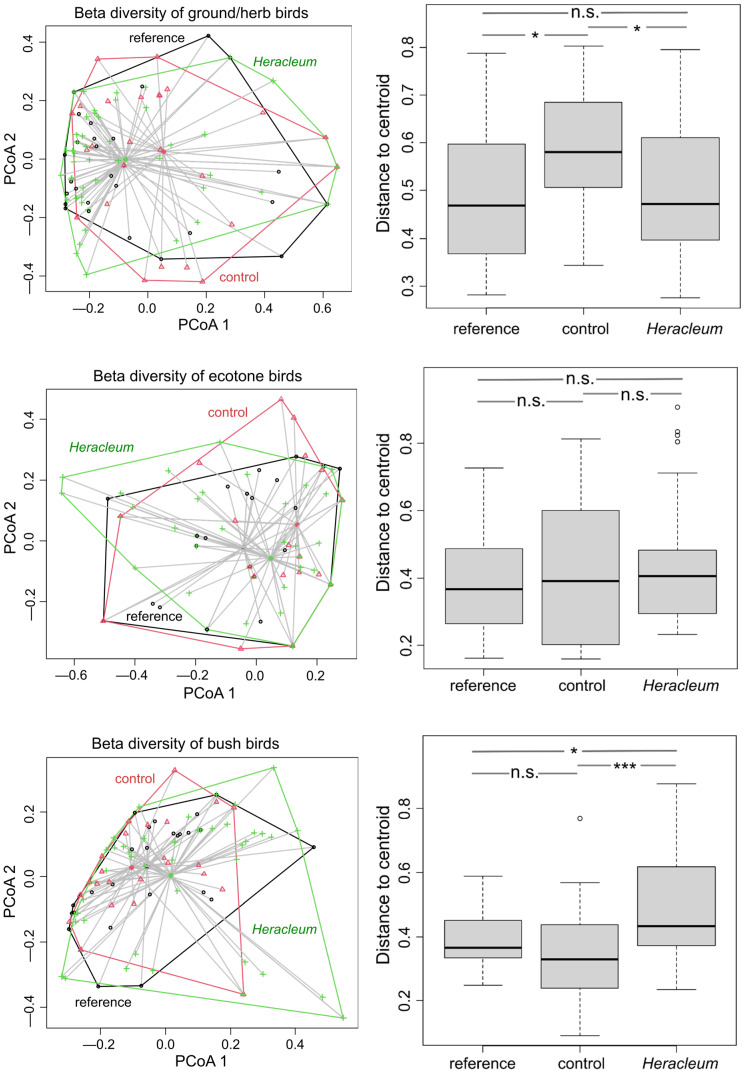

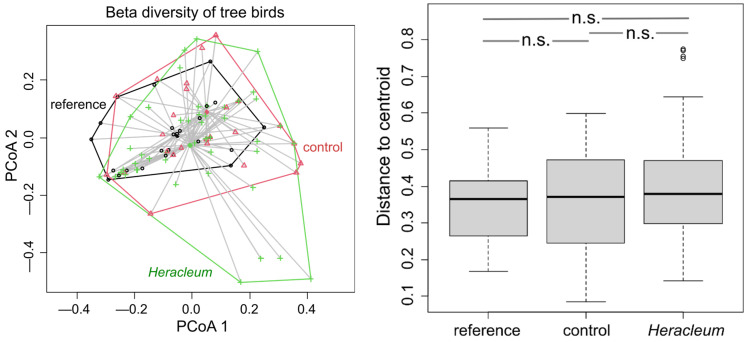
Scatter plots depicting the first two axes of the ordinations of bird species composition from assemblages and boxplots showing the distances of each site to the centroid, reflecting differences between sites in the compositions of birds (*p*-values: * < 0.05, *** < 0.001, n.s.—non-significant).

The PCoA ordination revealed divergence in the bird compositions among the invaded, control, and reference sites (One-way ANOVA: F = 4.929; *p* = 0.009), consisting of a gradual shift from the references to the controls and to the *Heracleum* sites (Figure 5). The differences between the reference and control sites were not significant (Tukey’s HSD, *p* = 0.717), with the same as between the controls and *Heracleum* sites (*p* = 0.109), and there were statistically significant differences between the references and *Heracleum* sites (*p* = 0.011). This pattern points to the presence of some exclusive species in the invasion-altered sites that were either not present or less frequent in uninvaded sites, in this case, the mentioned rook and barred warbler (see above), and was due to the absence of, e.g., some species using agricultural lands (Figure 1). There was also found segregation between sites in ground/herb dwellers and bush foragers, indicating dissimilar species compositions for these groups, due to the distinctive ground/herb dwellers’ composition on control sites and that of bush foragers on *Heracleum* sites (Figure 6).

In the case of ground/herb species, significant divergence between the sites was detected (One-way ANOVA: F = 3.699, *p* = 0.028), resulting from differences between the reference and control sites (Tukey’s HSD, *p* = 0.031) and the control and *Heracleum* sites (Tukey’s HSD, *p* = 0.044), with no differences between the reference and *Heracleum* (Tukey’s HSD, *p* = 0.756)—Figure 6. No divergence was found in the case of ecotone species between sites (One-way ANOVA: F = 0.61, *p* = 0.545). There were no differences between reference and control sites (Tukey’s HSD, *p* = 0.867), reference vs. *Heracleum* sites (*p* = 0.519), or *Heracleum* vs. control sites (*p* = 0.866). In the case of bush foragers, divergence was shown between the sites (One-way ANOVA: F = 9.748, *p* < 0.001), which resulted from differences between the control and *Heracleum* sites (*p* < 0.001) and the *Heracleum* vs. reference sites (*p* = 0.021), although there were no differences between the remaining reference and control sites (*p* = 0.409). There was no significant divergence in tree foragers between the sites (One-way ANOVA: F = 1.898, *p* = 0.155). There were no differences between the control and *Heracleum* sites (*p* = 0.306), reference and control sites (*p* = 0.979), or *Heracleum* vs. reference sites (*p* = 0.210)—Figure 6.

## 4. Discussion

The conducted research showed that invasion-induced habitat changes impacted both the species richness (i.e., alpha diversity) of individual bird assemblages and the divergence of assemblages between sites (i.e., beta diversity), confirming that plant invasions decrease the diversity of natives at least over small spatial scales [44]. However, in both cases, the extent of the invasion impact was different. The presence of hogweeds significantly reduced species richness in all studied assemblages compared to uninvaded (control) sites, despite the lacking decreases in bird abundances from the uninvaded to *Heracleum* sites, excluding ground/herb dwellers, whose number decreased. In terms of comparison at the level of habitat assemblages and beta diversity, the invaded sites were characterized by a unique composition of bush foragers compared to both uninvaded sites, confirming that anthropogenic activities (in this case, the introduction of plant invaders) create specific ecosystems [5]. The ground/herb dwellers at the control sites represented a distinct set of birds in comparison with other sites. This result indicates the validity of comparing the bird assemblage with reference sites, as it suggests an increase in the species diversity of ground/herb dwellers at the control site exposed to invasion compared to reference sites with possibly natural habitats. Research on assemblages according to the standard methodology with paired sites with the same habitats and in the same landscape sometimes does not make it possible to compare semi-natural and disturbed sites due to indirect effects related to the location of control sites in the landscape.

Comparative site analyses at the species level did not detect significant differences between reference and control sites or between *Heracleum* and reference sites. However, ordination analysis investigating the distribution of various species indicated that at least some habitat specialists were interchanging in space, e.g., reference sites included ground/herb dwellers that used agricultural lands, such as the ortolan bunting, but this bird was replaced by the related common reed bunting *Emberiza schoeniclus* on the remaining sites (Figure 1). This, however, confirmed that agricultural land use has a positive effect on species richness [45], because all sites were in the agricultural landscape and only the references contained the largest croplands, away from settlements. Another example was the presence of barred warblers mainly at the *Heracleum* sites and the preference of the common whitethroat *Sylvia communis* for the paired control and *Heracleum* sites, with an overall greater diversity of warblers from the genera *Sylvia* and *Phylloscopus* at reference sites (Figure 1). This means that although significantly lower bird species richness was found in all assemblages at invaded sites rather than control sites, all site types were characterized by a different set of species. This could have resulted from both deterministic and stochastic processes; in the first case, it would have been due to habitat properties [10], and in the second case, because of local colonization or the absence of specific species [21]. It could have been due to the effect of resource competition between similar species, because paired and reference sites were similar in habitats (Table A1 and Table A2); therefore, differences in environmental suitability may have been an unlikely reason for species turnover in the study system [46]. These results imply the important role of niche-based assembly processes in driving bird communities across multiple habitats [47]. 

When comparing habitats on invaded, control, and reference sites, they differed in the area of ruderal habitats and water availability (Table A1). Paired, i.e., invaded, and control sites, were characterized by a larger ruderal area, and *Heracleum* sites additionally contained a significantly larger water area (Table A1 and Table A2). This would explain the absence of some species using agricultural lands at paired sites despite their presence on reference sites (stochastic process), as ruderal habitats are rather degraded. Caucasian hogweeds being in the vicinity of water, e.g., rivers, is not surprising because this is the route of spread of these invaders [33]. However, it is worth noting the lack of differences in the areas of most habitat types between sites (Table A1). This confirms that the reduction in species diversity during invasion was due to its properties [33,35], i.e., it is a deterministic process. Because the bird species richness at the reference sites did not differ from that at the paired sites, only the ordination analysis detected stochastic episodes for some bird species. 

The conducted research confirmed that the filtering effect of vegetation is independent of losses and replacements of species [9], because in terms of species composition, the *Heracleum* and control sites were more similar to each other than to the reference sites (Figure 1). And yet despite this difference between paired sites, significant differences between them were detected in the number of species from all assemblages. It is worth adding that while DCA analysis (Figure 1) indicated a wider range of at least some species on reference sites than on paired sites, beta diversity analysis showed that invaded sites contained unusual lists of species (Figure 5). The negative impact of Caucasian hogweeds on species diversity in all bird assemblages confirmed that introduced non-native species are associated with lower taxonomic, functional, and phylogenetic diversity of communities and negatively impacts ecosystem functioning, which is particularly evident in habitats where human disturbance favors non-native species [48]. The results suggest that even within a generally human-modified landscape (here, including, e.g., control sites), invaded community diversity is always more affected by, and thus has a lower resilience to, disturbance. Thus, restoring and protecting natural habitats within human-modified landscapes is likely to increase the resilience of native species [48].

On the other hand, it must be stressed that the research approach in this paper was based on correlations and it was difficult to fully assess the mechanisms of the observed relationships between assembling birds and plant invasion. In terms of the generally lower diversity of native species in invaded communities, there were two possible effects: (1) invaders directly affected bird species, e.g., through the enhanced competition or particular habitat disturbance decreasing the number of habitat specialists (possible in ground/herb dwelling species, decreased in numbers and richness) and increasing the number of generalists (possible in ecotone birds, bush foragers, and tree species decreased in richness despite no reduction in the number from uninvaded towards invaded sites) and (2) sites with lower habitat and bird diversity could more likely host invading species, which contrasts with invaded sites being treated with caution, as the habitats there may have had low diversity even without invasion.

The results of this research confirmed that beta diversity provides valuable insights into the mechanisms driving biodiversity changes and their consequences for multiple ecosystem functions [49]. Differences in alpha diversity between paired sites were demonstrated quite surprisingly because, despite the similarities in species and habitats between them, which proves the properties of Caucasian hogweeds, it does not present the mechanisms of birds’ reaction to invasion. Beta diversity analysis, in turn, indicated that the invaded sites created a bush bird assemblage that was significantly different from the one on control sites, but also on the reference sites. The second detected phenomenon concerned the ground/herb dwellers’ assemblage which was distinct on control sites compared to other sites. These results show the mechanisms of the impact of invasion on birds. Invasive hogweeds morphologically resemble bushes and, therefore, have probably become attractive to these birds, but in a selective way depending on the species, shaping an unusual set of bush birds. In the case of ground/herb dwellers, their unusual grouping compared to other control sites may have resulted from the availability of herbaceous ruderal habitats (Table A2), and then, the rapid impact of invaders on the birds at *Heracleum* sites. These are the mechanisms of the impact of invasion on bird assemblages that suggest the implementation of specific management to preserve natural bird assemblages, i.e., hogweeds occurring in the vicinity of shrubs and herbal habitats and, e.g., grasslands being removed. Focusing on beta diversity is especially important in ecological communities that are subject to large environmental fluctuations and disturbances [49].

It is worth adding that at the regional scale, species diversity results mostly from changes in species composition among habitat patches, which only occur when multiple habitat areas are preserved. In fragmented landscapes, most of the habitat patches are small and individually host a low diversity. However, these patches often greatly differ in beta diversity and this heterogeneity can compensate for much of the local diversity loss, which works especially well in the case of forested areas [50]. Therefore, ecotone birds and tree foragers could also develop unusual associations in invaded areas, but in this case, it is not easy to change habitats because the availability of other forest patches is limited and often distant. The lack of an effect of invasion on tree and ecotone birds may have been undetected under beta diversity assumptions, although this effect was indicated by alpha diversity analysis.

## 5. Conclusions

The results indicated the impact of plant invasion on bird communities at the level of two habitat assemblages—ground/herb species loss from control to invaded sites and creating distinctive bush species compositions at invaded sites—despite losses in the species diversity and abundance of birds from all habitat assemblages. This was most likely due to the transformation of the grassland layer and the thickening of the shrub layer by invasive plants, which resemble them in morphology. Beta diversity analysis, which detected this effect, provided valuable insights into the mechanisms driving biodiversity changes and their consequences for multiple ecosystem functions. All site types (control, invaded, references) were characterized by a different set of species. This could have resulted from both deterministic and stochastic processes; in the first case, it would have been due to habitat properties, and in the second case, because of local colonization or the absence of specific bird species. The conducted research confirmed that the filtering effect of vegetation is independent of losses and replacements of species, because in terms of species composition, the *Heracleum* and control sites were more similar to each other than to the reference sites, and yet despite this difference between paired sites, significant differences between them were detected. 

## Data Availability

Data are available upon reasonable request.

## Appendix A

**Table A1:** Differences in the area of habitat variables between the control, *Heracleum*, and reference sites (in a radius of 100 m from counting points) tested by the Kruskal–Wallis test. Additionally, the habitats typical of open landscape (meadows, ruderal habitats) were summed up as “open areas”, while bushes and forests, as “overgrown areas”. Elements related to human activities (roads, buildings, agriculture) were taken together as “anthropogenic areas”.

Habitat Variable	Kruskal–Wallis Test			Dunn Test (Z; *p*-Value Adjusted)		
	χ^2^	df	*p*-Value	Control—*Heracleum*	Control—Reference	*Heracleum*—Reference
Meadows	5.08	2	0.079	2.15; 0.095	0.68; 0.498	−1.80; 0.107
Ruderal area	17.55	2	<0.001	−1.74; 0.081	2.10; 0.053	4.12; <0.001
Buildings	4.49	2	0.106	−0.06; 0.951	1.69; 0.135	1.76; 0.233
Agriculture	2.36	2	0.307	0.41; 0.683	−0.97; 0.495	−1.45; 0.445
Forests	0.31	2	0.854	−0.26; 0.796	0.26; 0.796	0.56; 0.578
Bushes	2.85	2	0.240	0.84; 0.604	1.68; 0.279	0.71; 0.475
Roads	1.59	2	0.450	−0.13; 0.898	0.95; 0.511	1.10; 0.814
Water	7.59	2	0.022	−1.26; 0.206	1.27; 0.307	2.73; 0.019
Open area	3.59	2	0.166	1.89; 0.176	0.99; 0.324	−1.20; 0.348
Overgrown area	2.06	2	0.357	−0.38; 0.706	0.91; 0.542	1.35; 0.532
Anthropogenic area	1.36	2	0.506	−0.14; 0.888	−1.03; 0.913	−0.86; 0.580
Number of patches	1.58	2	0.453	−0.17; 0.861	0.92; 0.539	1.12; 0.790

**Table A2:** Descriptive statistics of habitat variables between the control, *Heracleum*, and reference sites (in a radius of 100 m from counting points).

Habitat Variable	Control	*Heracleum*	Reference
	Mean ± SD, Range (ha)	Mean ± SD, Range (ha)	Mean ± SD, Range (ha)
	100 m Radius		
Meadows	1.686 ± 1.153, 0–3.140	1.012 ± 0.955, 0–2.647	1.485 ± 1.223, 0–3.140
Ruderal area	0.083 ± 0.150, 0–0.624	0.195 ± 0.391, 0–1.844	0.027 ± 0.077, 0–0.362
Buildings	0.038 ± 0.115, 0–0.547	0.063 ± 0.200, 0–0.970	0.002 ± 0.009, 0–0.054
Agriculture	0.376 ± 0.757, 0–2.545	0.362 ± 0.784, 0–2.704	0.787 ± 1.234, 0–3.140
Forests	0.568 ± 0.792, 0–2.430	0.602 ± 0.814, 0–2.791	0.641 ± 0.991, 0–3.016
Bushes	0.226 ± 0.267, 0–1.147	0.188 ± 0.230, 0–0.883	0.136 ± 0.151, 0–0.608
Roads	0.084 ± 0.110, 0–0.452	0.116 ± 0.191, 0–0.870	0.055 ± 0.064, 0–0.209
Water	0.078 ± 0.173, 0–0.500	0.081 ± 0.163, 0–0.547	0.008 ± 0.027, 0–0.124
Open area	1.769 ± 1.082, 0–3.140	1.208 ± 0.934, 0–2.647	1.512 ± 1.234, 0–3.140
Overgrown area	0.795 ± 0.837, 0–2.649	0.789 ± 0.767, 0–2.791	0.777 ± 1.037, 0–3.061
Anthropogenic area	0.450 ± 0.823, 0–2.726	0.478 ± 0.839, 0–2.793	0.842 ± 1.236, 0–3.140
Number of patches	8.143 ± 4.672, 1–16.000	8.429 ± 4.694, 2–20.000	7.286 ± 4.827, 1–25.000
Invasion area	–	0.520 ± 0.617, 0.015–2.270	

## Appendix B

R script concerning beta diversity (accompanying Figure 5 and Figure 6)

> library(betapart)

> library(vegan)

> levels(df$group)

[1] “control”   “Heracleum” “reference”

> groups <- factor(df$group, labels = c(“reference”,”control”,”Heracleum”))

> df1<-ifelse(df>0,1,0)

> df2 <- subset(df1, select = c(2:81))—all species

> dist<-beta.pair(df2, index.family=“jaccard”)

> bd<-betadisper(dist[[3]],groups)

> plot(bd)

> anova(bd)

> TukeyHSD(bd)
